# Comparison between surgical fusion and the growing-rod technique for early-onset neurofibromatosis type-1 dystrophic scoliosis

**DOI:** 10.1186/s12891-020-03460-6

**Published:** 2020-07-11

**Authors:** Siyi Cai, Liqiang Cui, Guixing Qiu, Jianxiong Shen, Jianguo Zhang

**Affiliations:** Department of Orthopaedic Surgery, Peking Union Medical College Hospital, No.1 Shuai Fu Yuan, Wang Fu Jing Street, Beijing, Post Code: 100730 China

**Keywords:** Neurofibromatosis type-1, Dystrophic early-onset scoliosis, Posterior fusion, Growing rod

## Abstract

**Background:**

Spinal deformities constitute one of the most common types of manifestations of neurofibromatosis type-1 (NF-1), which can lead to either dystrophic or non-dystrophic early-onset scoliosis (EOS). Surgical treatment for EOS with NF-1 is challenging, and the outcomes have rarely been reported. The anterior-posterior procedure is widely used, but posterior-only fusion is theoretically easier and safer to perform. Is it possible that a new surgery that accommodates growth is a better choice? A direct comparison between posterior fusion and growth-friendly surgery in terms of surgical outcomes has not yet been conducted in dystrophic EOS with NF-1 patients.

**Methods:**

Baseline information was extracted from the NF-1 database at our institute with approval from the local ethics committee. All enrolled patients were diagnosed with NF-1. Clinical and radiographic data were recorded preoperatively, after the initial surgery, and at the final follow-up. Implant-related, alignment, neurological complication and unplanned revision surgery data were recorded. We compared the outcomes of these two groups in terms of curve correction, growth parameters, complications and unplanned revision surgeries.

**Results:**

There were eight patients in the PF group and eight patients in the GR group, with a mean follow-up of 51.0 ± 17.5 months. The main curve size was similar (PF 67.38° ± 17.43° versus GR 75.1° ± 26.43°, *P* = 0.501), and there were no significant differences in the initial surgery correction rate or the rate of correction. However, the patients in the GR group exhibited more T1-S1 growth during the follow-up overall and per year than did those in the PF group. The operative time was significantly longer for the PF group than for the GR group (PF, 4.39 ± 1.38 vs. GR, 3.00 ± 0.42 h; *p* = 0.008). Significantly fewer segments were involved in the PF group (8.25 ± 3.20) than in the GR group (13.00 ± 1.60).

**Conclusion:**

For the initial treatment of dystrophic EOS in patients with NF-1, the GR technique is possibly a more appropriate treatment than is the PF technique in terms of trunk growth. However, the repeated procedures required for GR may be a considerable disadvantage. More studies with direct measurement of pulmonary function must be conducted to determine the effect of GR on pulmonary development. More studies with larger sample sizes and longer follow-up periods are needed to fully assess the treatment strategies.

## Background

Neurofibromatosis type-1 (NF-1), first described by von Recklinghausen in 1882, is a rare autosomal dominant neurocutaneous disorder. It is caused by a mutation in chromosome 17q11.2 of the NF-1 gene, which leads to a loss of function of the neurofibromin protein. This results in a wide variety of manifestations and clinical complications since it affects most organ systems, including deterioration of the skin, bones (neurofibroma), arteries, peripheral nerves, and the central nervous system. NF-1 has an incidence of 1 in 2500 to 3000 individuals and a prevalence of 1 in 4000 to 5000 individuals [1, 2]. It has been reported that 10–60% of NF-1 patients present with early-onset spinal deformities, leading to either dystrophic or non-dystrophic scoliosis [3]. Typical dystrophic NF-1 scoliosis has a short, sharp curve and can be recognized by three or more of the following features: rib pencilling, vertebral scalloping, wedging, rotation, and spindling of the transverse process.

Considering the nature of dystrophic NF-1 scoliosis, which has a tendency of curve progression, leading to poor pulmonary function and truncal height loss, fusion is usually recommended [4–6]. Generally, the more severe the dystrophic changes are, the higher the likelihood of a scoliotic curvature. Dystrophic scoliosis used to be treated aggressively, and anterior-posterior fusion has been widely used [7, 8]. In recent years, to avoid encountering extensive plexiform tumours during the anterior approach, posterior-only fusion (PF) has been reportedly used in treating NF-1 dystrophic scoliosis, yielding good short-term outcomes [9, 10]. However, other than in one recent report [11], the long-term outcomes of early posterior spinal fusion in patients aged ≤10 years old have not been well reported.

Growth guidance using the growing-rod (GR) system is an alternative surgical treatment that has been demonstrated to be safe and effective in treating early-onset scoliosis (EOS). However, its use and outcomes in NF-1 patients have not been extensively reported in the literature [12, 13]. The purpose of this study was to evaluate and compare the medium-term surgical results and complications between these two posterior-based surgical techniques in early-onset NF-1 dystrophic scoliosis patients.

## Methods

### Patients

**Fig. 1:**
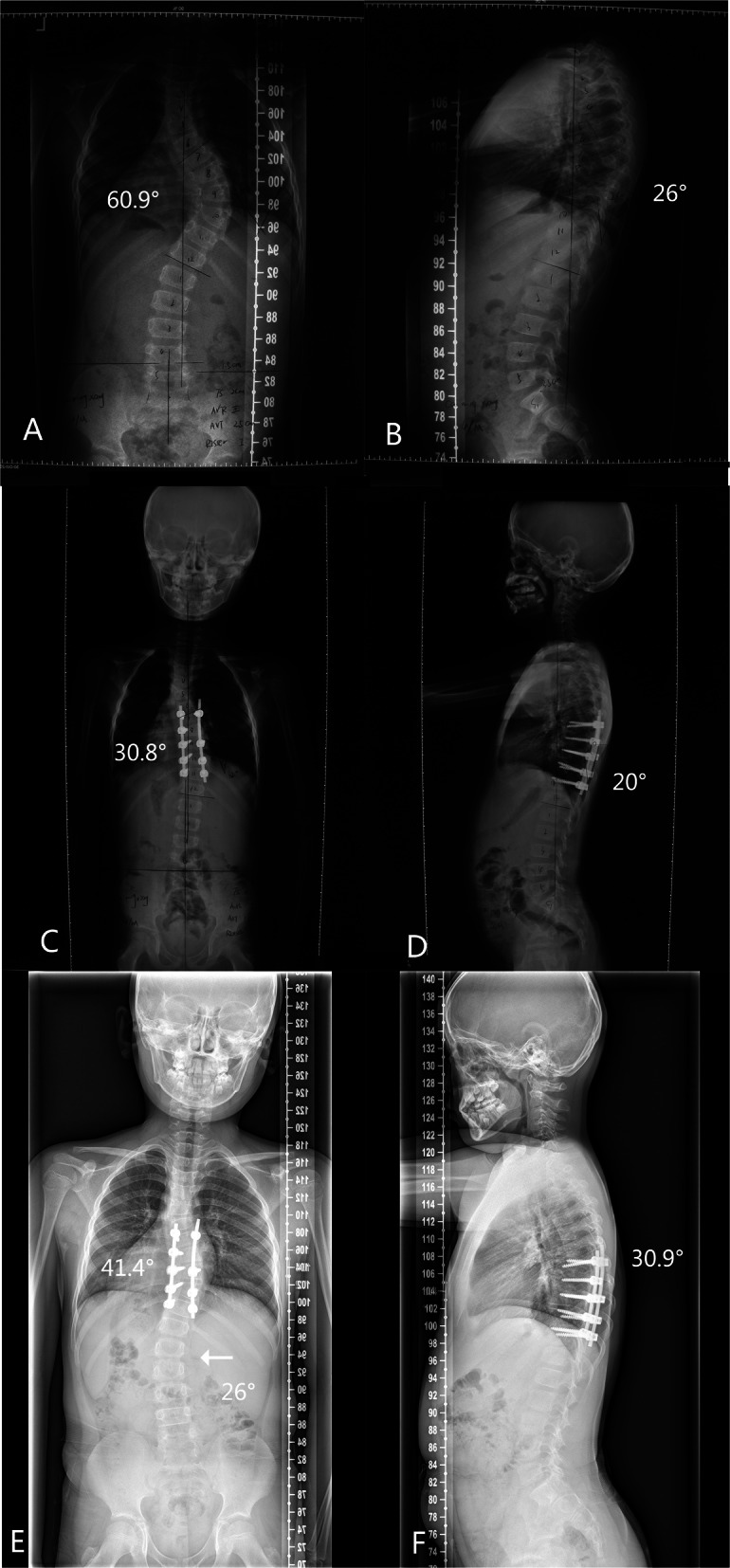
Case 2 in PF group: Radiographs of a 4-year-old patient with neurofibromatosis and a 60.9°right thoracic scoliosis, who received posterior only fusion operation from T7- T11. **a**. **b**. Preoperation. **c**. **d**. Postoperation. **e**. **f**. The adding on phenomenon (arrow) was obvious at the 34-month follow-up, which was attributed to at the growth of the anterior column of the fusion segments

A retrospective review of all NF-1 patients who underwent primary surgical treatment for scoliosis between March 2008 and March 2015 was conducted. The inclusion criteria were as follows: 1) early-onset dystrophic scoliosis with NF-1 and an age of ≤10 years at the initial surgery; 2) main curve in the thoracic region; and 3) a minimum follow-up of ≥2 years. Patients were excluded if they underwent combined anterior-posterior surgery or if they were lost to follow-up. NF-1 was diagnosed on the basis of established criteria [1]. Dystrophic scoliosis was diagnosed on the basis of the criteria used in Lykissas’s article [14] (Fig. 1).

### Clinical and radiographic data

Medical records were reviewed, and the following clinical data were retrieved from the NF-1 database of our institute: patient demographics, surgical details (including the surgical segments involved), the types of anchor instrumentation (hook, screw, or hybrid), diameters of the rod, bone grafting strategy (material, location), intraoperative neurophysiology monitoring, operative time, blood loss, and the frequency of lengthening for the GR system.

In terms of the radiographic data, Cobb angles for the major coronal curve and the sagittal T5-T12 kyphotic curve, the T1-S1 length, and proximal and distal junctional kyphotic curves were measured preoperatively, after the initial surgery, and at the final follow-up. Preoperative MRI scans were performed for all patients to screen for tumours and evaluate the conditions of the neural structures.

All complications were recorded, and the following were reviewed: implant-related aspects (rod breakage, dislodgment or prominence, screw malposition, or fracture), alignment complications (fusion curve progression or crankshaft phenomenon, sagittal or coronal trunk decompensation, proximal or distal junctional kyphosis), and neurological complications (transient or permanent neurological deficits or worsening).

Unplanned procedures were defined as unscheduled surgical procedures performed to manage a complication, including revision surgeries in fusion cases. Planned procedures were defined as procedures that were scheduled as part of the routine GR treatment protocol. The crankshaft phenomenon was defined as the progression of the Cobb angle by ≥10° [15]. Proximal junctional kyphosis was defined as a sagittal Cobb angle > 10° measured between the inferior endplate of the uppermost instrumented vertebra (UIV) and the superior endplate of the vertebra 1 level above the UIV. Distal junctional kyphosis was defined as a sagittal Cobb angle > 10° measured between the superior endplate of the lowermost instrumented vertebra (LIV) and the inferior endplate of the vertebra 1 level below the LIV [16].

All radiographic measurements were performed on a computer by two experienced spine surgeons working independently. The surgeons were blinded to the measured outcomes during the study, and the mean values were used for analysis.

### Surgical procedures

None of the patients underwent preoperative traction. Pedicle screws were the preferred type of instrumentation in all cases. When the pedicle was not large enough or was obscured, hybrid instrumentation using a hook and screws was utilized. The upper and lower instrumented vertebrae were the neutral vertebrae.

In the PF group, the deformity was corrected by a combination of rod derotation and sequential in situ translational reduction, with or without in situ bending of the rod. Additional correction manoeuvres, including appropriate compression and/or distraction, were applied to correct the deformity in three dimensions. The posterior elements were decorticated, and bone grafts were placed on the decorticated bed using autogenous local bone grafts in combination with allogenic bone grafts. In two cases, the concave-side paraspinal tumours were resected. Postoperative hard bracing was prescribed for 6–8 months for all patients.

In the GR group, the standard dual-rod technique, as described by Akbarnia et al., was used in all cases [17, 18]. The proximal and distal foundation implants used were pedicle screws or a combination of pedicle screws and hooks. The foundation sites were fused with autogenous local bone grafts mixed with allogenic bone grafts. The rod and the connector were placed underneath the deep fascia. To control the apical shift, three pedicle screws were placed in the apical sites on the convex side in two cases.

### Statistical analysis

The data were analysed using SPSS (IBM Corp. Released 2013. IBM SPSS Statistics for Windows, Version 19.0. Armonk, NY: IBM Corp.) and expressed as the mean ± SD. The patients were divided into two groups according to whether they underwent PF or GR. For the independent samples, Student’s t-test and the Mann–Whitney U test were used to compare the normally and nonnormally distributed data, respectively. The chi-square test was used to compare the complication rate and the unplanned surgery rate. A value of *p* < 0.05 was considered statistically significant.

## Results

Twenty-six patients with NF-1 scoliosis were identified in our database. Among these, we excluded patients who underwent anterior-posterior fusion (3 cases, 11.5%), those who underwent one-stage posterior osteotomy with short segment fusion (2 cases, 7.7%), and those whose follow-up period was < 2 years (3 cases, 11.5%). Two patients in the PF group whose major curve was in the lumbar region were excluded based on the second inclusion criterion. Thus, eight cases of PF and eight cases of GR were included in our study. The average age of the patients (3 males, 5 females) in the PF group was 7.7 ± 2.3 years, and the mean follow-up was 56.9 ± 18.2 months. The average age of the patients (2 males, 6 females) in the GR group was 7.4 ± 1.4 years, with a mean follow-up of 51.0 ± 17.5 months. The mean age (*p* = 0.74), sex ratio (*p* = 0.58), and follow-up duration (*p* = 0.52) were similar between groups.

### Surgical data

The operative time was longer for the PF group than for the GR group (4.39 ± 1.38 vs. 3.00 ± 0.42 h; *p* = 0.008). Significantly fewer segments were involved in the PF group (8.25 ± 3.20) than in the GR group (13.00 ± 1.60). However, the number of instrumented segments was similar between the two groups (PF, 5.50 ± 1.93 vs. GR, 4.63 ± 1.19, *p* = 0.262). The details on the implants placed in the apical vertebrae and the rod diameters are listed in Table 1. In the PF group, eight initial procedures and one revision surgery were performed. In the GR group, a total of 55 procedures, including eight initial operations and 47 lengthening surgeries, four of which were unplanned revision surgeries, were performed. An average of 5.88 ± 1.13 lengthening surgeries were performed per patient in the GR group, and the interval between these surgeries ranged from 6 to 11 months. 
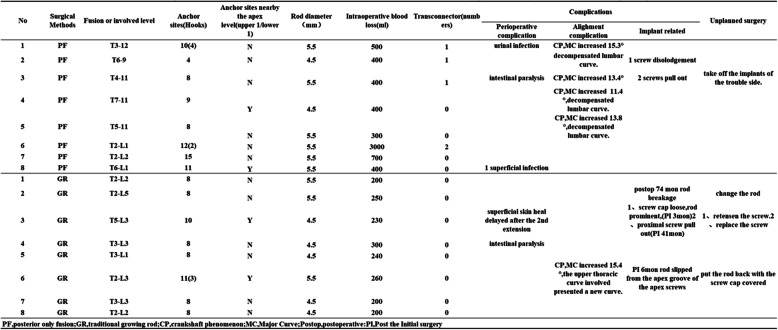

**Table 1:** Clinical Data and surgical information on 16 NF-1 Patients With Early-onset Scoliosis Treated by posterior only fusion or traditional growing rods in our center

No	Surgical Methods	Fusion or involved level	Anchor sites(Hooks)	Anchor sites nearby the apex level(upper 1/lower 1)	Rod diameter(mm)	Intraoperative blood loss(ml)	Transconnector(numbers)	Complications			Unplanned surgery
								Perioperative complication	Alighment complication	Implant related	
1	PF	T3-12	10(4)	N	5.5	500	1	urinal infection	CP,MC increased 15.3°		
2	PF	T6-9	4	N	4.5	400	1		decompensated lumbar curve.	1 screw disolodgement	
3	PF	T4-11	8	N	5.5	400	1	intestinal paralysis	CP,MC increased 13.4°	2 screws pull out	take off the implants of the trouble side.
4	PF	T7-11	9	Y	4.5	400	0		CP,MC increased 11.4°,decompensated lumbar curve.		
5	PF	T5-11	8	N	5.5	300	0		CP,MC increased 13.8°,decompensated lumbar curve.		
6	PF	T2-L1	12(2)	N	5.5	3000	2				
7	PF	T2-L2	15	N	5.5	700	0				
8	PF	T6-L1	11	Y	5.5	400	0	1 superficial infection			
1	GR	T2-L2	8	N	5.5	200	0				
2	GR	T2-L5	8	N	5.5	250	0			postop 74 mon rod breakage	change the rod
3	GR	T5-L3	10	Y	4.5	230	0	superficial skin heal delayed after the 2nd extension		1、screw cap loose,rod prominent,(PI 3mon)2、proximal screw pull out(PI 41mon)	1、retensen the screw.2、replace the screw
4	GR	T3-L3	8	N	4.5	300	0	intestinal paralysis			
5	GR	T3-L1	8	N	4.5	240	0				
6	GR	T2-L3	11(3)	Y	5.5	260	0		CP,MC increased 15.4°,the upper thoracic curve involved presented a new curve.	PI 6mon rod slipped from the apex groove of the apex screws	put the rod back with the screw cap covered
7	GR	T3-L3	8	N	4.5	200	0				
8	GR	T2-L2	8	N	4.5	200	0				

*PF* posterior only fusion, *GR* traditional growing rod, *CP* crankshaft phenomenon, *MC* Major Curve, *Postop* postoperative, *PI* Post the Initial surgery

### Curve correction and growth gained

The mean preoperative Cobb angle of the primary curve was 67.4 ± 17.4° in the PF group and 75.1 ± 26.4° in the GR group (*p* = 0.61). The initial correction rate was 52.1 ± 15.3% in the PF group and 56.5 ± 11.9% in the GR group (*p* = 0.44). During the follow-up, the major curve of 3 patients in the GR group decreased; all others showed a fair degree of progression. Four patients in the PF group exhibited major curve progression. However, there were no significant differences between the two groups in terms of the mean correction rate loss (the initial correction rate (%) - the final correction rate (%): PF, 11.5 ± 8.2% vs. GR, 4.2 ± 11.7%; *p* = 0.20). The preoperative, initial postoperative, and final follow-up vales for the T5-T12 kyphotic curve and the T1-S1 distance are presented in Table 2. The patients in the GR group exhibited more T1-S1 growth per year than did those in the PF group (gain/year; 11.7 ± 2.6 vs. 5.6 ± 1.7 mm; *p* = 0.00). 
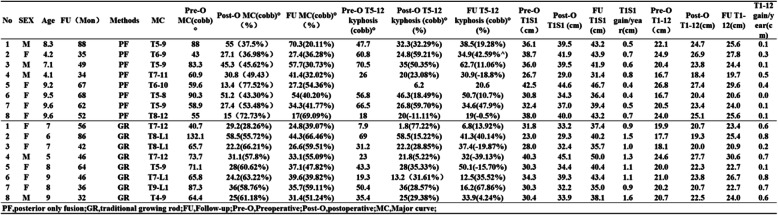

**Table 2:** Clinical and radiographic Data of 16 NF-1 Patients With Early-onset Scoliosis Treated by posterior only fusion or traditional growing rods

No	SEX	Age	FU(Mon)	Methods	MC	Pre-O MC(cobb)°	Post-O MC(cobb)°(%)	FU MC(cobb)°(%)	Pre-O T5-12 kyphosis (cobb)°	Post-O T5-12 kyphosis (cobb)°(%)	FU T5-12 kyphosis (cobb)°(%)	Pre-O T1S1(cm)	Post-O T1S1 (cm)	FU T1S1 (cm)	T1S1 gain/year(cm)	Pre-O T1-12(cm)	Post-O T1-12(cm)	FU T1-12(cm)	T1-12 gain/year(cm)
1	M	8.3	88	PF	T5-9	88	55 (37.5%)	70.3 (20.11%)	47.7	32.3 (32.29%)	38.5(19.28%)	36.1	39.5	43.2	0.5	22.1	24.7	25.6	0.1
2	F	4.2	35	PF	T6-9	43	27.1 (36.98%)	27.4 (36.28%)	60.8	24.8 (59.21%)	34.9(42.59%^)	38.7	41.9	43.9	0.7	24.9	26.9	27.8	0.3
3	M	7.1	49	PF	T5-9	83.3	45.3 (45.62%)	57.7 (30.73%)	70.5	35 (50.35%)	62.7(11.06%)	36.0	39.5	41.9	0.6	20.4	23.8	24.4	0.1
4	M	4.1	34	PF	T7-11	60.9	30.8 (49.43)	41.4 (32.02%)	26	20 (23.08%)	30.9(-18.8%)	26.7	29.0	31.4	0.8	16.7	18.4	19.7	0.5
5	F	9.2	67	PF	T6-10	59.6	13.4 (77.52%)	27.2 (54.36%)		6.2	20.6	42.5	44.6	46.7	0.4	26.8	27.4	29.6	0.4
6	F	9.5	68	PF	T5-8	90.3	51.2 (43.30%)	54 (40.20%)	56.8	46.3 (18.49%)	50.7(10.7%)	30.8	34.3	36.4	0.4	16.7	20.4	20.6	0.0
7	F	9.6	62	PF	T5-9	58.9	27.4 (53.48%)	34.3 (41.77%)	66.5	26.8 (59.70%)	34.6(47.9%)	32.4	37.0	39.4	0.5	20.5	23.4	24.0	0.1
8	F	9.6	52	PF	T8-12	55	15 (72.73%)	17 (69.09%)	18	20 (-11.11%)	19(-0.5%)	38.0	40.0	43.2	0.7	24.0	25.1	25.6	0.1
1	F	7	56	GR	T7-12	40.7	29.2 (28.26%)	24.8 (39.07%)	7.9	1.8 (77.22%)	6.8(13.92%)	31.8	33.2	37.4	0.9	19.9	20.7	23.4	0.6
2	F	6	86	GR	T8-L1	132.1	58.5 (55.72%)	44.3 (66.46%)	69	58.5 (15.22%)	41.3(40.14%)	23.0	29.3	40.2	1.5	17.7	19.3	25.4	0.8
3	F	7	42	GR	T8-L1	65.7	22.2 (66.21%)	26.6 (59.51%)	31.2	22.2 (28.85%)	37.4(-19.87%)	28.0	32.4	35.7	1.0	18.1	20.0	20.9	0.2
4	M	5	46	GR	T7-12	73.7	31.1 (57.8%)	33.1 (55.09%)	23	21.8 (5.22%)	32(-39.13%)	40.3	45.1	50.0	1.3	24.6	27.7	30.6	0.7
5	F	8	64	GR	T5-9	71.1	28 (60.62%)	37.1 (47.82%)	43.3	28 (35.33%)	50.1(-15.70%)	30.3	34.4	40.4	1.1	20.0	22.3	22.7	0.1
6	F	9	46	GR	T7-L1	65.8	24.2 (63.22%)	39.6 (39.82%)	19.3	13.2 (31.61%)	12.5(35.52%)	34.3	39.3	43.4	1.1	21.0	23.8	26.7	0.8
7	F	8	36	GR	T9-L1	87.3	36 (58.76%)	35.7 (59.11%)	50.4	36 (28.57%)	16.2(67.86%)	30.3	32.2	35.0	0.9	20.2	20.7	22.7	0.7
8	M	9	32	GR	T4-9	64.4	25 (61.18%)	31.4 (51.24%)	35.4	25 (29.38%)	33.9(4.24%)	30.4	33.9	38.1	1.6	20.7	22.5	24.0	0.6

*PF* posterior only fusion, *GR* traditional growing rod, *FU* Follow-up, *Pre-O* Preoperative, *Post-O* postoperative, *MC* Major curve

### Complications

Three cases in the PF group had general perioperative complications (one urinary tract infection, one postoperative ileus, and one superficial wound infection). Two patients in the GR group had perioperative complications (one delayed wound healing after the second distraction and one postoperative ileus). There were no statistically significant differences between the two groups in terms of the incidence of general complications (Table 1). There were no neurological complications in any of the patients.

**Fig. 2:**
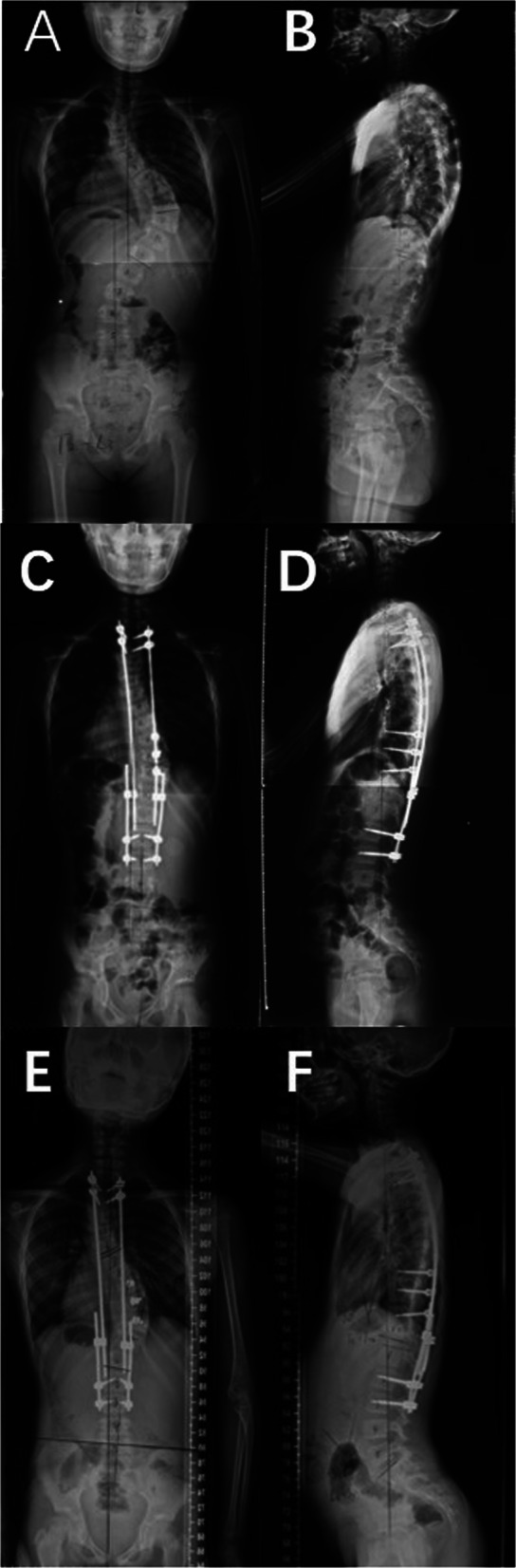
Case 6 in GR group: Radiographs of a 9-year-old patient with neurofibromatosis and a 65.8°right thoracic scoliosis, who received posterior Growing Rod Correction from T2- L3 and 3 pedicle screws without caps were putted in apex area. A.B. Preoperation. C.D. Postoperation. E.F. The rod of the right side slipped from the apex groove 6 months later from the initial surgery. The major curve increased 15.4°, the upper thoracic curve was increased

**Fig. 3:**
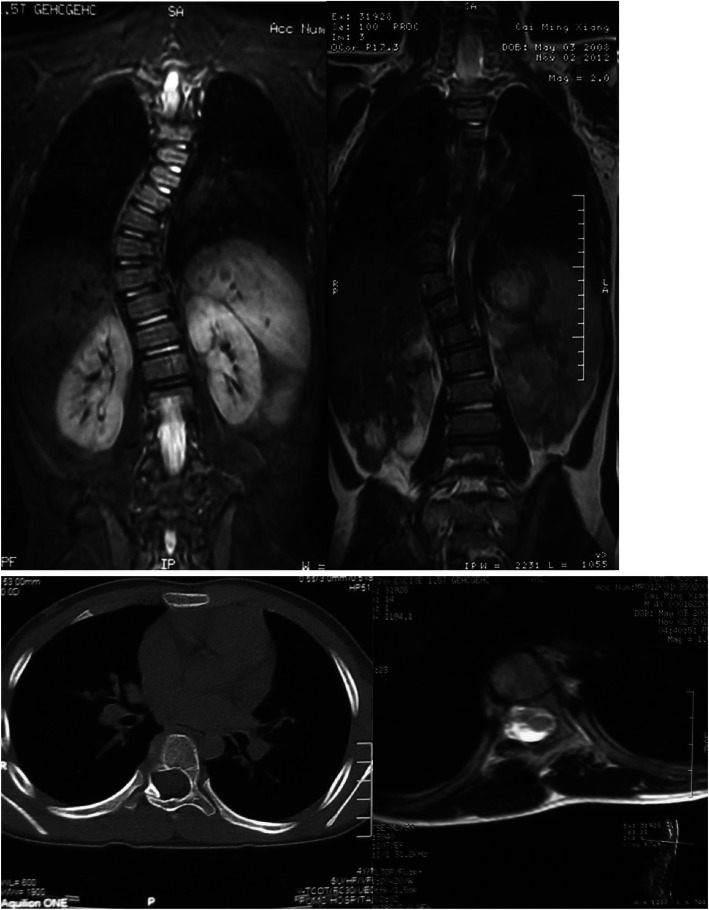
We attached which is the CT and MRI of Case 2. For the coronary image of MRI, we can see high signal of T2WI on the concave of the vertebrae, which may be the structure of neurofibroma. For the axial images of CT, enlarged vertebral canal and tapered pedicle of vertebral arch can be seen. Meanwhile, there are more 6 typical pigmentation spot of the patient’s skin. Diagnosis of all the patients meet the clinical diagnostic criteria as well

Radiographically, five patients (62.5%) in the PF group experienced alignment complications during the follow-up period: four of them experienced curve deterioration of the fused segments by for more than 10^o^, and one experienced decompensation, with a new lumbar curve (Fig. 2). On the other hand, only one patient in the GR group experienced alignment complications; the upper thoracic structural curve within the instrumented segments deteriorated by 15.4^o^ (Fig. 3). This difference was statistically significant between the two groups (*p* = 0.026), indicating that the GR group had fewer alignment complications. There were no cases of proximal or distal junctional kyphosis in any of these patients. There were two implant-related complications in the PF group and four in the GR group.

## Discussion

In this study, we reported the clinical outcomes of the posterior-only surgical treatment for dystrophic EOS in patients with NF-1. This is the first study to compare the outcomes of PF to GR surgery in dystrophic NF-1 scoliosis patients under the age of 10 years.

For cases of non-dystrophic EOS, treatment strategies similar to those for idiopathic scoliosis can be used [19]. Regarding dystrophic EOS cases, for which the deformity progresses rapidly [14], conservative treatment cannot control progression, and surgical intervention is often indicated [5, 6]. Surgical treatments for NF-1 are challenging to perform [20]. A few cohort studies [11, 12, 21, 22] have indicated that combined anterior-posterior fusion is recommended for EOS with NF-1. However, anterior-posterior fusion requires a more complicated operation and has a higher risk. Currently, pedicle screw-based instrumentation allows better control of the vertebral body than do Harrington rods or hook-rod-based instrumentation, thus making PF possible. PF also does not require access through the thoracic/abdominal cavity, which can reduce the difficulty and risk of complications associated with surgery. Recent studies have shown that PF surgery can yield good outcomes [10, 19]. Given that major curves can progress despite being fused, early fusion can cause inadequate thoracic growth in NF-1 patients. Therefore, delaying fusion has been recommended [23]. The GR system can delay fusion and reduce the operative time and blood loss. Thus, it has been performed increasingly more often since its development. Both PF and GR methods have been reported to be used in the treatment of scoliosis, but no systematic reviews comparing these two approaches have been reported. On the other hand, the law of diminishing returns with growth rods should not be ignored, and it should be considered that either revision surgery or definitive fusion may be more difficult to perform after growth-rod treatment.

In our comparative study, the initial curve correction rates for these two groups (PF, 52.7%; GR, 56.47%) were similar to those reported for Jain’s GR group (59%) but did not reach the rate for the anterior-posterior fusion group (66.15%) [11, 12]. Additionally, the number of segments instrumented was similar between our study in Jain’s study. In most cases, the progression of scoliosis is caused by pseudarthrosis. For patients with EOS, the crankshaft phenomenon is another important complication [24]. It was reported that 21% of EOS patients with NF-1 who underwent PF experienced the crankshaft phenomenon during the follow-up period in Greggi’s study [21]. GR reduces the occurrence of the crankshaft phenomenon, as does the posterior procedure, while allowing the trunk to elongate with growth. In our GR group, one in eight patients experienced the crankshaft phenomenon, and the follow-up correction rate remained at 52.27%, which is similar to that in the series reported by Jain et al. (50.8%) [12]. We should also be aware that decompensation in the fusion group can be related to the level selected for fusion.

The GR group favourably demonstrated T1-S1 growth and T1-T12 growth, whereas the PF group did not. In our study, the mean number of fused segments was 8.25 per patient in the PF group; the PF group showed significantly less T1-S1 growth than did the GR group, and the GR group demonstrated approximately 1.12 cm/year of growth in Jain’s series [12]. An increase in the height from T1-T12, the main parameter of the thoracic cavity, may be essential for lung growth and pulmonary function [25]. The T1-T12 growth/year in the GR group (0.60 ± 0.27 cm/year) was also significantly greater than that in the PF group (0.20 ± 0.15 cm/year) (*p* = 0.02), indicating that for EOS patients with NF-1, the GR approach is better than the PF approach in terms of thoracic cavity growth. Karol et al. mentioned in their study that patients with proximal thoracic deformity who require early fusion are at risk for restrictive pulmonary disease. The pursuit of alternative procedures to treat early spinal deformity is merited [26]., Yang et al. mentioned in their review that growth-friendly spine surgery has been shown to correct spinal deformity while allowing growth of the spine and subsequently lung growth [27]. Karol et al. also mentioned in their article that diminished thoracic spinal height correlates with decreased forced vital capacity [28]. We believe more studies with direct measurement of pulmonary function in GR group patient must be conducted to determine the effect of GR on pulmonary function development.

In the PF group, two patients had proximal screw dislodgment (one was displaced, and one was pulled out), one of whom underwent revision. In the GR group, one patient suffered loosening of the cap of the proximal screw, causing the rod to loosen and come out during the interval between lengthening surgeries. In the study by Jain et al., proximal construct failure was the most common implant-related complication (5/14) [12]. The correction force is high and the bone mineral density is low in EOS patients with NF-1, which are the main reasons for proximal anchor site failure. Early aggressive treatment, such as surgeries for dystrophic EOS in NF-1 patients, has been recommended. In Greggi’s report and our study, the clinical results of PF were unsatisfactory due to the high occurrence of deformity progression and its limitation of thoracic growth. The GR system, however, retained the possibility of growth and allowed fusion to be delayed in patients with NF-1 while leading to a degree of deformity progression similar to that of PF. In our study, the GR group had better outcomes in terms of blood loss, operative time, correction maintenance, and spinal trunk growth than did the PF group. However, it is noteworthy that the need for repeated surgeries was a disadvantage for the GR group in our study. This disadvantage may be improved by the newer version of magnetically controlled GRs. This newer version of GR with noninvasive distraction has been successfully used for EOS cases of various aetiologies [29, 30]. Though its efficacy in the context of NF-1 patients is currently unknown, it is reasonable to expect better outcomes with magnetically controlled GR with regard to the number of scheduled follow-up surgeries.

This study has some limitations. First, this is a retrospective study; thus, it is difficult to perform a strictly case-matched comparison. As it is a fusion technique, anterior-posterior fusion can lead to more reliable fusion outcomes than can PF. It would have been better to use the anterior-posterior fusion technique in a control group to determine the superiority of the GR technique in treating dystrophic EOS in patients with NF-1. Second, the follow-up duration was not long enough to observe the effects of different techniques on EOS patient outcomes and allow a comprehensive analysis of the cases in these children. Third, it can be argued that PF is a complete therapy, while GR is an incomplete therapy because of the need for repeated surgeries. For our study, we assessed the outcomes from the time of the initial surgery, as we intended to focus on the initial surgical treatment choice. If the efficacy of the magnetically controlled GR technique is established, it is worthwhile to compare it with PF. We did not have direct pulmonary assessment to measure pulmonary function data for all the patients, and the use of T1-S1 gain as a proxy for pulmonary function improvement is only shown as a reference but was not directly indicated. And the sample size in our study was small, and it was similar to that in the study by Akbarnia et al. [17] the statistical power of our study is relatively low because EOS with NF-1 is a relatively rare condition, and it is difficult to include a large number of patients. If possible, a multi-centre study with a larger sample size should be conducted to further investigate the problem and help us understand more about this condition.

## Conclusions

Overall, after comparing the abovementioned parameters and clinical outcomes between the PF group and the GR group, we concluded that GRs and PF are both suitable options for treatment for dystrophic EOS in patients with NF-1, and the GR system, which allows more trunk growth, may be more appropriate for the initial treatment. More studies with direct measurement of pulmonary function must be conducted to determine the effect of GR on pulmonary development. More studies with larger sample sizes and longer follow-up periods are needed to fully assess the treatment strategies.

## Supplementary information

**Additional file 1: Figure S1.** Only one patient had decompensation with a new lumbar curve, it could related to the fusion level selection rather than the method of treatment.

## Data Availability

The datasets used and/or analysed during the current study are available from the corresponding author on reasonable request.

## Abbreviations

EOS: Early-onset scoliosis
NF-1, GR: Growing rod; Neurofibromatosis type-1
PF: Posterior-only fusion
