# Supplementary material for: Comparison between surgical fusion and the growing-rod technique for early-onset neurofibromatosis type-1 dystrophic scoliosis
Source: BMC Musculoskelet Disord. 2020 Jul 11;21:455. doi: 10.1186/s12891-020-03460-6 (PMC7354683; doi:10.1186/s12891-020-03460-6)
Supplement: Supplementary file 1 — Additional file 1: Figure S1. Only one patient had decompensation with a new lumbar curve, it could related to the fusion level selection rather than the method of treatment. [file 12891_2020_3460_MOESM1_ESM.docx]

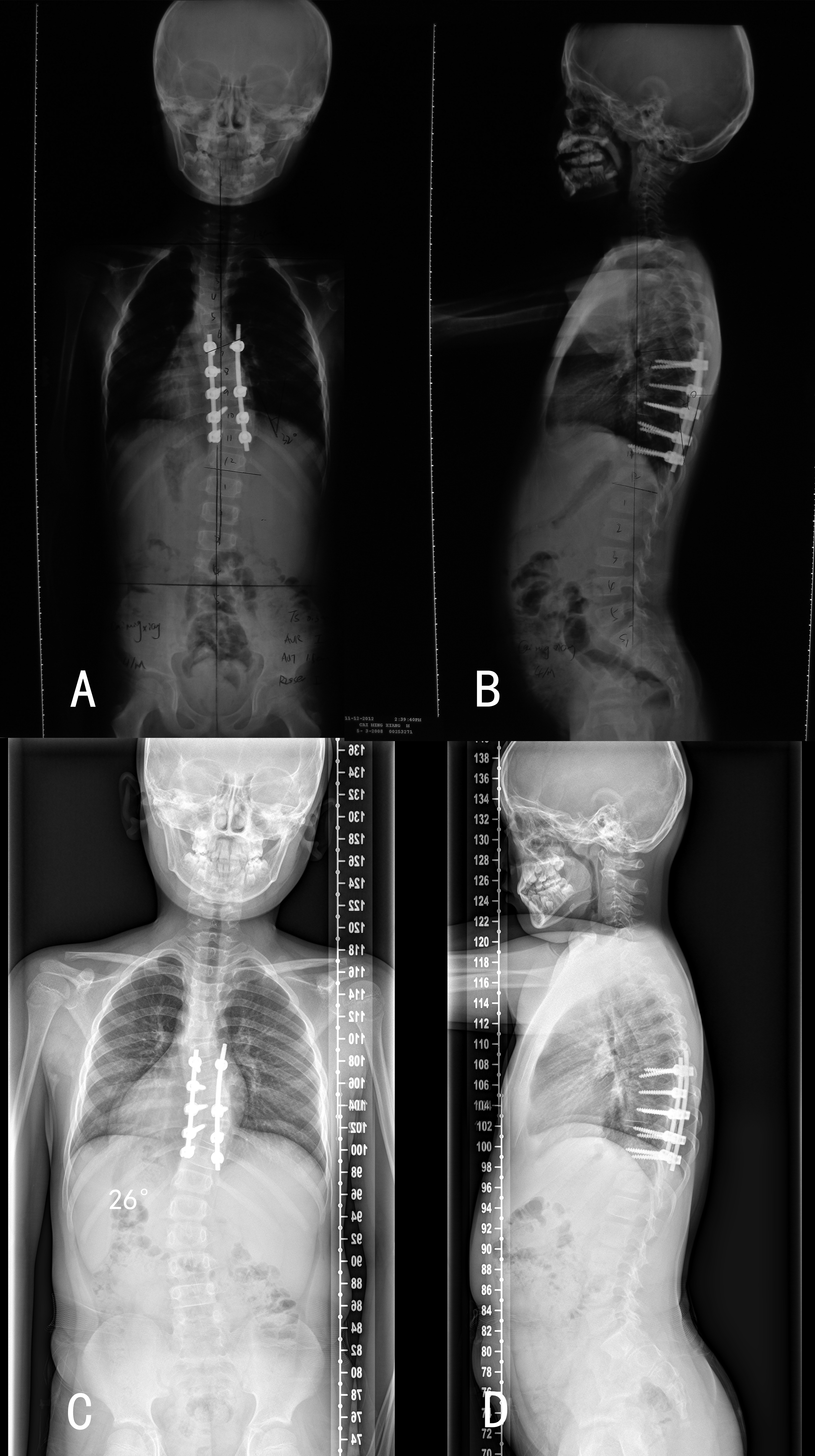


Only one patient had decompensation with a new lumbar curve, it could related to the fusion level selection rather than the method of treatment
